# P02-008 - Dramatic response to canakinumab in MKD

**DOI:** 10.1186/1546-0096-11-S1-A115

**Published:** 2013-11-08

**Authors:** B Makay, E Ünsal

**Affiliations:** 1 Pediatric Rheumatology, Dokuz Eylül University Hospital, İzmir, Turkey

## Introduction

Mevalonate kinase deficiency-associated periodic fever syndrome (MKD) is a systemic autoinflammatory disease caused by mutations in the mevalonate kinase gene (MVK), previously named "hyper-IgD syndrome" due to its characteristic increase in serum IgD level. The patients suffer recurrent fever attacks every 2-8 weeks beginning from infancy, often precipitated by immunizations, infections or emotional stress. Fever lasts 2-7 days and can be accompanied by malaise, headache, diarrhea, abdominal pain, vomiting, skin rashes, arthralgia, arthritis, tender lymphadenopathy and hepatosplenomegaly.

Fever attacks usually respond to the administration of steroids. However, increasing frequency of fever episodes with steroid use and the natural chronic disease course may require a continuous long-term treatment. Colchicine, cyclosporine, thalidomide and statins are not effective. A TNF-α blocking agent etanercept and an IL-1 blocking agent anakinra have been demonstrated to reduce the frequency of fever attacks in MKD. Canakinumab is a human monoclonal antibody targeted at interleukin-1 beta. Here, we report a 6-year-old boy with MKD who had a dramatic response to canakinumab.

## Case report

The patient was diagnosed as MKD when he was 2-year-old based on two mutations in MVK gene (Exon 8. c.803T>C (p.268I>T), and Exon 10 c.1129 G>A (p.377V>I). Colchicine, simvastatin and etanercept failed to reduce the attacks. He could not tolerate daily anakinra injections after a trial of two months, though a favourable response. Canakinumab, another IL-1 blocker, was started at a dose of 4 mg/kg every 28 days, which had a dramatic response, and he has never had attack since then.

## Discussion

Canacinumab may be a therapeutic option in mevalonate kinase deficiency-associated periodic fever syndrome.

## Disclosure of interest

None declared.

